# Improving Performance of Clinical Research: Development and Interest of Electronic Health Records

**DOI:** 10.1155/2015/864549

**Published:** 2015-10-12

**Authors:** Ariel Beresniak, Andreas Schmidt, Danielle Dupont, Mats Sundgren, Dipak Kalra, Georges J. E. De Moor

**Affiliations:** ^1^Data Mining International, Geneva, Switzerland; ^2^Andreas Schmidt with Innovation Management, Product Development, F. Hoffmann-Laroche Ltd., Basel, Switzerland; ^3^Data Mining America, Montreal, Canada; ^4^AstraZeneca, Molndal, Sweden; ^5^University College London, London, UK; ^6^University of Ghent, Ghent, Belgium

Collecting health information from patients is probably one of the most ancient medical acts. The Hippocratic Corpus was a compendium of medical records from Ancient Greece and one of the first attempts to classify diseases according to symptoms and observations.

Over the years, health records have been mainly used in paper format to document individual patient characteristics, to keep track of treatments, and to report patient outcomes. Nowadays, as medical care is getting more and more complex and personalized, the recent advances and uses of information technologies enable capturing, processing, storing, and mining patient-level data in order to quickly extract meaningful clinical information and medical knowledge for clinical decision making and to further personalize health care. With their widespread use, Electronic Health Records (EHRs) have become a very important tool from which new services can be provided. This major breakthrough is already starting to transform research and development of innovative health products by enhancing and speeding up existing processes. This is because EHR data can be used to rapidly optimise clinical protocol designs, to better identify and faster recruit eligible patients for clinical trials, and to foster new, efficient ways of collecting data during clinical study conduct and improve the detection and reporting of serious adverse events.

Despite available technologies, numerous challenges remain which still require significant efforts and time: ethical, legal, data privacy issues, information technology systems integration, optimal interoperability for a seamless and trustworthy data exchange, and so forth.

Importantly, major innovations such as developing best in class and seamless EHR-enabled clinical data exchange systems require building awareness and trust with multiple stakeholders in order to maximize the expected benefits, as well as societal acceptance from patients, health care providers, governmental bodies, and clinical trial sponsors. In particular, clinical research investigators and sponsors have long been using conventional clinical research processes that rely more upon ad hoc case finding and ploughing through large numbers of paper records. They will thus require special attention, including the development and dissemination of customized value propositions that explain and evidence innovative EHR-based clinical research platforms, how these compare with existing practices, and which qualitative and quantitative benefits they can deliver in order to facilitate adoption and large scale implementation.

EHRs offer an unprecedented opportunity, as well as technological challenges, to change the current clinical research paradigm, including the following:

1. Patient databases are growing rapidly and are becoming more accessible.
2. The diversity of health records is important, covering all kinds of populations and health conditions.
3. EHRs are heterogeneous, which makes interoperability (seamless data exchange) and integration of information a challenging task.
4. For a particular reuse of EHRs, only a selection of key parameters (data items) might be useful for clinical research.
5. EHRs are dynamic: the monitoring of the changes could become important features for many research applications.
6. The use of EHR data for clinical research could speed up the patient recruitment phase, reduce the number of protocol amendments, improve the efficiency of major parts of the clinical trial process, and reduce costs.
7. EHRs offer many opportunities for data mining, such as to extract original meaningful information from a large set of patients or populations.

Given the growing demand worldwide for clinical evidence (including from real-world contexts), as well as the formidable challenges in clinical research today (including costly protocol amendments, significant delays in patient recruitment, time-consuming and redundant clinical data entry in appropriate data management systems, and the escalation in research and development costs), the question is not so much if clinical research will use these new concepts in current practice and benefit from reusing EHR data, but when.

As more and more applied research domains are now exploring how to use electronic platforms to facilitate key clinical research tasks, and considering the inexorable trend towards modernising clinical research models to create, deliver, and capture more value and benefits, it appears to be good timing to provide a scientific overview on the most advanced research and developments in this field in the frame of a special issue.

This special issue provides an opportunity to present the latest scientific contributions and technological developments in this emerging field that can be derived from the research use of EHR data.

The objectives of this special issue are twofold:

1. For the first time, to bring together and to present some of the latest research and development efforts in this field, including technological R&D, surveys, and pilot studies.
2. To support more and focused research activities in this domain. Given the large number of clinical trials worldwide, the next challenge will naturally be to ensure a seamless and sustainable deployment of these advanced and trustworthy interoperable platforms in order to enable the reuse of EHR data at the global level.

There is no doubt that the original articles published in this special issue already evidence this emerging reality for the future of clinical research, thanks to the contributions of a broad number of highly experienced and specialized authors in this field. The presented articles address a wide range of perspectives and compile the most promising research findings and the latest developments.

As EHRs will continue to have a significant and positive impact on state-of-the-art clinical development, we are confident that this special issue will stimulate further ideas and research for enhancing, speeding up, and optimising clinical research worldwide, towards delivering effective and safe innovative medicines to health care faster, to the benefits of patients, the entire health systems, and society.

*Ariel Beresniak* *Andreas Schmidt* *Danielle Dupont* *Mats Sundgren* *Dipak Kalra* *Georges J. E. De Moor*

